# Genetically altering the expression of neutral trehalase gene affects conidiospore thermotolerance of the entomopathogenic fungus *Metarhizium acridum*

**DOI:** 10.1186/1471-2180-11-32

**Published:** 2011-02-10

**Authors:** Yajun Leng, Guoxiong Peng, Yueqing Cao, Yuxian Xia

**Affiliations:** 1 Genetic Engineering Research Center, College of Bioengineering, Chongqing University, Chongqing, 400030, China; 2 Chongqing Engineering Research Center for Fungal Insecticides, Chongqing, 400030, China; 3 Key Lab of Functional Gene and Regulation Technologies under Chongqing Municipal Education Commission, Chongqing, 400030, China

## Abstract

**Background:**

The entomopathogenic fungus *Metarhizium acridum *has been used as an important biocontrol agent instead of insecticides for controlling crop pests throughout the world. However, its virulence varies with environmental factors, especially temperature. Neutral trehalase (*Ntl*) hydrolyzes trehalose, which plays a role in environmental stress response in many organisms, including *M. acridum*. Demonstration of a relationship between *Ntl *and thermotolerance or virulence may offer a new strategy for enhancing conidiospore thermotolerance of entomopathogenic fungi through genetic engineering.

**Results:**

We selected four *Ntl *over-expression and four *Ntl *RNA interference (RNAi) transformations in which *Ntl *expression is different. Compared to the wild-type, *Ntl *mRNA expression was reduced to 35-66% in the RNAi mutants and increased by 2.5-3.5-fold in the over-expression mutants. The RNAi conidiospores exhibited less trehalase activity, accumulated more trehalose, and were much more tolerant of heat stress than the wild-type. The opposite effects were found in conidiospores of over-expression mutants compared to RNAi mutants. Furthermore, virulence was not altered in the two types of mutants compared to the wild type.

**Conclusions:**

*Ntl *controlled trehalose accumulation in *M. acridum *by degrading trehalose, and thus affected conidiospore thermotolerance. These results offer a new strategy for enhancing conidiospore thermotolerance of entomopathogenic fungi without affecting virulence.

## Background

*Metarhizium acridum *is a haploid entomopathogenic fungus (Hypocreales: Clavicipitaceae). *M. acridum *isolates have been used as biocontrol agents for crop pests, including sugar cane grubs, termites, cockroaches, and rhinoceros beetles [1]. *M. acridum *was commercialized and used for locust control in Australia, West Africa [2], and China [3].

Insecticide resistance, pest resurgence, and concerns over environmental impact have made the search for alternative means of biological pest control more urgent. Unfortunately, large-scale use of fungal biocontrol agents is partially limited by the failure of conidia to retain virulence during long-term storage, transportation, and use under stressful conditions, such as high temperature, low humidity, and sunlight exposure [4-6]. Manipulation of culture conditions could optimize the concentration of spore polyols and sugars, including trehalose, and consequently increase tolerance to low relative humidity [7,8]. However, genetic manipulations of these polyols and sugars to enhance environmental tolerance have not been explored in entomopathogenic fungi.

To genetically engineer more robust entomopathogenic fungi, we focused on the trehalose pathways involved in stress response. Trehalose is a storage carbohydrate as trehalose concentrations are high when nutrients are limited in resting cells. In many microorganisms and invertebrate animals, trehalose plays a role in environmental stress response [9,10] and is a known stress metabolite as its concentration increases during certain adverse environmental conditions, such as exposure to heat or toxic chemicals [11]. In *Saccharomyces cerevisiae*, trehalose is required for cells to survive diverse stresses, such as heat shock, starvation, and desiccation [12]. Additionally, it has been shown to provide one way for cells to survive thermal stress *in vitro *[13]. Based on the stress-protection properties of trehalose *in vitro *and the positive correlation between trehalose concentration and stress resistance *in vivo*, it is reasonable to expect that trehalose might function as a protective agent against stress [14,15].

However, studies investigating the relationship between trehalose and thermotolerance have shown conflicting results. In *S. cerevisiae*, the trehalose level was positively correlated with stress resistance in different strains, growth conditions, and heat treatments [16-18]. Almost all strains exhibited more than a 2- to 10-fold increase in trehalose level after heat-shock treatment [19,20]. Additionally, the defective mutant of the neutral trehalase gene (*Ntl) *produced organisms that were more thermotolerant than the wild type, most likely because of higher trehalose levels [21]. In contrast, some studies found no correlation between trehalose accumulation and thermotolerance under certain conditions, suggesting that trehalose may not mediate thermotolerance [22,23].

In most fungal species, trehalose hydrolysis is carried out by trehalase [24]. The single known exception is *Pichia fermentans*, in which trehalase has phosphorylase activity [25]. Fungal trehalases are classified into two categories according to their optimum pH: acid trehalases or neutral trehalases [26,27]. Cytosolic neutral trehalase degrades intracellular trehalose. The *Ntl *of *S. cerevisiae*, *Kluyveromyces lactis*, *Candida utilis*, *Torulaspora delbrueckii*, *Schizosaccharomyces pombe*, and *Pachysolen tannophilus *is tightly controlled by signaling pathways that end with the trehalose being reversibly activated by phosphorylation [27]. These signaling pathways can be triggered *in vivo *by glucose, nitrogen sources, heat shock, and chemicals like protonophores, which produce intracellular acidulation. This enzyme has been thoroughly studied in filamentous fungi, such as *Aspergillus nidulans*, *Neurospora crassa*, and *Magnaporthe grisea *[21,28], but little is known about *M. acridum *neutral trehalase (*Ntl*) beyond the sequence in two strains, *M. roberstii *ARSEF2575 [29,30] and CQMa102 [31]. Using these sequences and genetic manipulation tools, we can now determine how *Ntl *affects stress response in terms of thermotolerance and virulence.

Different fungal growth phases (budding, conidiation, and germination) are associated with trehalose accumulation or mobilization. Depletion of trehalose storage marks early germination of fungal spores [26]. In *Cryptococcus neoformans *and other pathogenic fungi, the trehalose pathway is a selective fungicidal target for antifungal development [28,32]. It is not known whether *Ntl *is a virulence factor in *M. acridum*.

We report here the construction of RNA interference (RNAi) and over-expression mutants of *Ntl *to investigate its role in thermotolerance and virulence of *M. acridum*. The results offer a new strategy for improving the thermotolerance of fungal conidia and yield insights into *M. acridum *spore physiology.

## Results

### Over-expression and RNA interference mutants and the expression of *Ntl*

**Figure 1:**
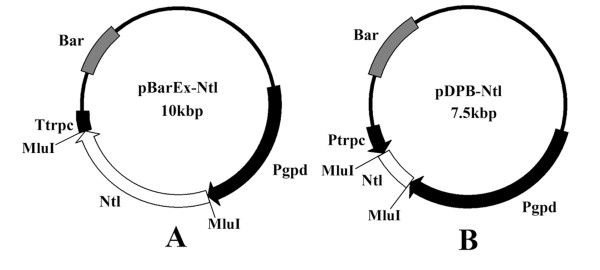
Schematic diagram of the *Ntl *over-expression vector (A) and the *Ntl *RNAi vector (B)

The pBarEx-NTL over-expression vector contained a 2,535-nucleotide sequence from the *Ntl *genomic DNA fragment, including the full coding sequence and parts of the promoter and terminator sequences (Figure 1A). The pDPB-NTL vector contained 435 nucleotides of the *Ntl *coding sequence (Figure 1B). Both constructs were transformed to *M. acridum *CQMa102 using microparticle bombardment. Four *M. acridum *transformants for each construct were selected according to their ability to grow on selective media. PCR analysis showed that the vector was integrated into the fungal genome.

**Figure 2:**
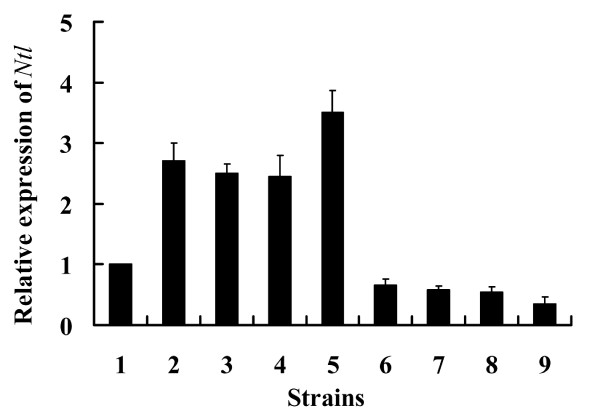
**Real-time PCR analysis for relative expression of *Ntl***. 1: wild-type strain; 2-5: over-expression mutants; 6-9: RNAi mutants. *Gapdh *was analyzed in parallel as a loading control (not shown). Standard error (SE) bars are averages for three independent experiments.

Expression of *Ntl *was analyzed by real-time PCR (Figure 2). In over-expression transformants, *Ntl *levels were 2.5-3.5-fold higher than in wild-type levels. In contrast, *Ntl *expression in RNAi transformants was reduced to 35-66% of wild-type levels.

### *Ntl *is related to trehalose accumulation in conidia

The neutral trehalase activity of conidia increased significantly in over-expression mutants compared to the wild-type strain and was reduced significantly in RNAi mutants (p < 0.05) (Table 1). Significantly positive correlation (correlation coefficient = -0.816, p < 0.05) was established between neutral trehalase activity and *Ntl *expression levels (Table 2). In contrast, the trehalose concentration in the wild-type strain was significantly higher than that in the over-expression mutants and lower than that in the RNAi mutants (P < 0.05). This showed that the neutral trehalase activity varied inversely with the trehalose concentration in conidia. Furthermore, the trehalose concentration was significantly positively correlated with *Ntl *expression levels and neutral trehalase activity (p < 0.05) (Table 2). This demonstrated that *Ntl *is related to trehalose accumulation because it controls the neutral trehalase activity.

**Table 1:** Trehalose concentrations and neutral trehalase activity in wild-type strain compared to over-expression mutants and RNAi mutants

Strains	Trehalose (pg/conidium)*		Neutral trehalase activity (U/mg protein)*	
1	7.17 ± 0.93	c	14.28 ± 1.14	c

2	5.04 ± 1.17	e	18.08 ± 1.15	ab

3	6.10 ± 0.22	d	16.43 ± 1.21	b

4	5.91 ± 0.27	de	16.29 ± 1.15	b

5	5.51 ± 0.53	e	16.12 ± 0.96	b

6	9.72 ± 0.14	b	8.82 ± 1.26	d

7	10.76 ± 0.83	a	7.59 ± 0.99	e

8	10.38 ± 0.83	ab	8.33 ± 1.12	de

9	10.57 ± 1.31	ab	8.23 ± 1.39	de

*Means (±SE) of 3 repetitions followed by different lowercase letters in the same column were significantly different at the p < 0.05 level according to the ANOVA table and Tukey's multiple range test. 1: wild-type strain; 2-5: over-expression mutants; 6-9: RNAi mutants.

**Table 2:** Correlation coefficients (R) of treatments and cellular components

	*Dry-heat(R)*	*Wet-heat(R)*	*Trehalose(R)*	*mRNA(R)*
mRNA	-0.9818	-0.890	-0.831	1.000
Trehalose	0.873	0.898	1.000	-0.831
Trehalase	-0.889	-0.905	-0.867	0.816

### *Ntl *affects conidiospore thermotolerance

**Figure 3:**
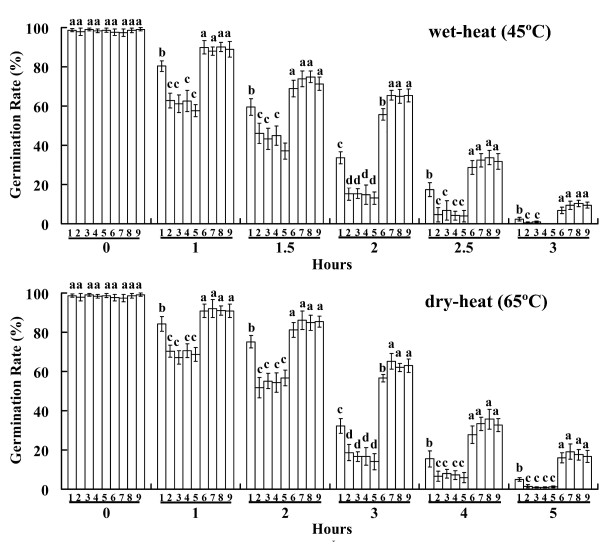
**Germination rates of *M. acridum *wild-type strain and *Ntl *mutants**. Wet-heat: aqueous conidial suspensions exposed to 45°C for 0, 0.5, 1, 1.5, 2, or 2.5 hours; dry-heat: dried conidia exposed to 65°C for 0, 1, 2, 3, 4, or 5 hours. 1: wild-type strain; 2-5: over-expression mutants; 6-9: RNAi mutants. Standard error bars (SE) show averages for three independent experiments. Significant differences are designated by the lowercase letters on the bars of each group (p < 0.05).

**Figure 4:**
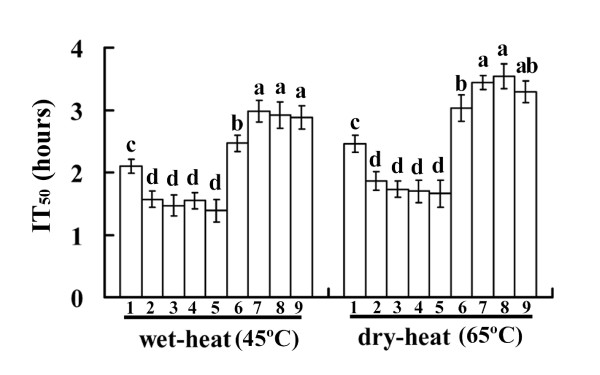
**IT**_**50 **_**of *M. acridum *wild-type strain and *Ntl *mutants**. IT_50_: inhibition time values for 50% germination of aqueous conidial suspensions exposed to 45°C and dried conidia exposed to 65°C, respectively. 1: wild-type strain; 2-5: over-expression mutants; 6-9: RNAi mutants. Standard error (SE) bars show averages for three independent experiments. Significant differences are designated by the different lowercase letters on the bars of each group in the wet-heat or dried-heat test (p < 0.05).

After wet-heat exposure at 45°C, the germination rate of conidia declined with increasing exposure time and the conidia germination rates of the wild-type strain and mutants appeared to be significantly reduced for each succeeding 0.5-hour interval (Figure 3). However, the response to tolerance was obviously different for the wild-type strain, over-expression mutants, and RNAi mutants. The conidia germination rate of the wild-type strain was significantly higher than that of the over-expression mutants (p < 0.05) and lower than that of the RNAi mutants (p < 0.05). Similar results were observed after dry-heat exposure at 65°C for 0, 1, 2, 3, 4, or 5 hours. Accordingly, the inhibition time value for 50% germination (IT_50_) of the wild-type strain was longer than that of the over-expression mutants (p < 0.05) and shorter than that of the RNAi mutants (p < 0.05) (Figure 4). These data showed that the *Ntl *over-expression mutants were significantly more sensitive to heat compared with the wild-type strain (p < 0.05). Contrary to that of the over-expression mutants, the thermotolerance of the *Ntl *RNAi mutants was significantly higher than that of the wild-type strain (p < 0.05).

Furthermore, both trehalase and *Ntl *mRNA levels were negatively correlated with the germination rates of conidia treated with wet heat and dry heat (p < 0.05) (Table 2), suggesting that *Ntl *affects conidiospore thermotolerance.

### *Ntl *has no effect on virulence

**Figure 5:**
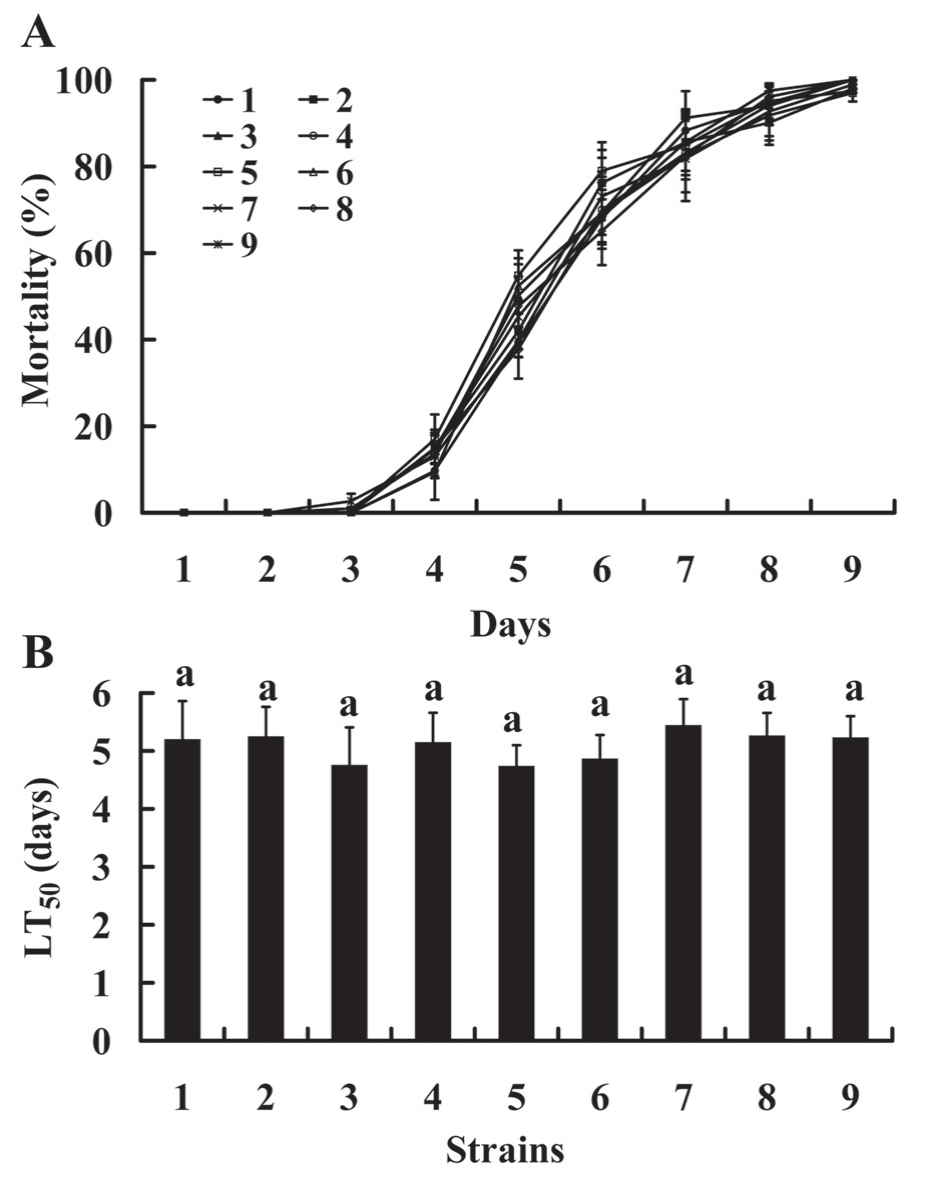
**Bioassay results for *M. acridum *against *Locusta migratoria***. 1: wild-type strain; 2-5: over-expression mutants; 6-9: RNAi mutants. A: mortality (±SE) of *Locusta migratoria *treated with wild-type strain and various *Ntl *transformants; B: lethal time values for 50% mortality (LT_50_) values of *Locusta migratoria *treated with wild-type strain and various *Ntl *transformants. Standard error (SE) bars are averages for four independent experiments. Same lowercase letters indicate no significant differences (p > 0.05).

Bioassays revealed that mortality trends of locusts inoculated with over-expression mutants or RNAi mutants were similar to that of locusts inoculated with wild strain (Figure 5A). Accordingly, no significant differences were observed in locust lethal time values for 50% mortality (LT_50_) between the wild-type strain, over-expression mutants, or RNAi mutants (p > 0.05) (Figure 5B). This result suggested changes in *Ntl *expression level did not affect the virulence of *M. acridum*.

## Discussion

Resisting thermal stress is important for pathogens of the locust, like *M. acridum*, because temperatures fluctuate in locust habitats and locusts themselves could also employ behavioral fever to counter fungal infection [33]. *Ntl *has been reported to play an important role in environmental stress response. In this study, the function of *Ntl *with respect to thermotolerance in *M. acridum *was investigated by changing its expression level via RNAi and over-expression mutants.

Trehalose is an important factor determining thermotolerance in *M. acridum*. Trehalose content and thermotolerance were significantly and positively correlated, and *Ntl *activity was significantly and negatively correlated with thermotolerance (Table 2). These results suggest that trehalose accumulation and metabolism play important roles in thermotolerance, but this factor is not the only controller of thermotolerance [22,34]. The accumulation and metabolism of other polyols, such as sucrose and glycerol, may also be factors in stress response [22]. It is possible that changes in trehalose concentration produced by up- or down-regulating trehalase levels may also affect the levels of other polyols and the entire metabolic process. Further investigation of other polyols in the *Ntl *mutants is required to understand fully the mechanism of the effect of *Ntl *on *M. acridum *thermotolerance.

Field conditions and abiotic environmental factors, such as temperature, moisture, and sunlight, influence whether infection can occur. When the host temperature favors a short germination time and that temperature is above or below the pathogen's optimum, temperature can be a limiting factor for the disease. However, oil-based formulations and selective media have been shown to enhance the thermotolerance of *M. acridum *conidia, resulting in promising acridid control in the field [35,36]. Using the genetic manipulation tools introduced here for *M. acridum*, the thermotolerance of the mycoinsecticidal strain will be improved to allow for wider commercial application.

A secretary trehalase activity of *M. acridum *was detected in the hemolymph of infected insects, suggesting that it is a virulence factor in insect pathogenesis [29]. In contrast, the changes in neutral trehalase expression had no effects on virulence in this study, which agrees with the report on *C. neoformans *that a neutral trehalase mutant does not possess any known virulence defects [32]. Our results indicate that trehalose in conidia does not affect virulence; thus, genetically engineering the trehalose pathway would increase the thermotolerance of fungal strains with no loss of virulence. Temperature tolerance also affects fungal agent storage longevity [4]. Further studies are required to investigate the longevity of the mutants.

The dual promoter RNAi system developed in this study successfully knocked down the gene expression in filamentous fungus. In previous studies, genes that were knocked down with *isopliae *over-expression and RNAi *Ntl *transformants exhibited no loss in virulence compared to wild-type silencing vectors that produced hairpin or intron-containing hairpin RNA in fungi [37-43], which involved two steps of oriented cloning. The dual promoter system simplified the RNAi construction procedure to one single-step non-oriented cloning, in which transcription of a target gene from each promoter produced a pool of sense and antisense RNAs in the cells. This system provides an easy and efficient tool for knocking down gene expression, and can be extended to knock down multiple gene targets from transcriptionally fused genes. Thus, the dual promoter system offers an efficient platform for functional analysis of entomopathogenic fungal genes and genetic manipulation for strain improvement.

## Conclusions

Our study shows that *Ntl *expression of *M. acridum *can be effectively enhanced or inhibited by over-expression or RNAi mutants, respectively, using a dual promoter system. Compared to the wild-type, *Ntl *mRNA was reduced to 35-66% in RNAi mutants and increased by 2-3-fold in the over-expression mutants. The conidiospores of RNAi mutants had less trehalase activity, accumulated more trehalose, and were much more tolerant of heat stress than the wild type. The opposite effects were found in conidiospores of over-expression mutants compared to RNAi mutants. The *Ntl *mRNA level was positively correlated with neutral trehalase activity and negatively correlated with trehalose concentration and the thermotolerance of conidiospores, further confirming the role of *Ntl *in the thermotolerance of *M. acridum*. Furthermore, bioassays showed that alteration of *Ntl *expression did not affect the virulence.

In conclusion, *Ntl *regulates thermotolerance through trehalose accumulation in *M. acridum *but does not affect its virulence. The use of the RNAi mutant of *Ntl *could provide a new strategy for improving the conidiospore thermotolerance of an entomopathogenic fungus without compromising its virulence.

## Methods

### Strain growth conditions

*M. acridum *strain CQMa102, a locust-specific strain, was isolated by our laboratory in Chongqing, China. Conidia were harvested from cultures grown on 1/4 strength Sabouraud's dextrose agar medium (SDA: 1% dextrose, 0.25% mycological peptone, 2% agar, and 0.5% yeast extract) at 28°C. Mycelia for DNA and RNA extraction were grown by inoculating 100 mL 1/4 SDA liquid media with 10^6 ^conidia and incubating at 28°C with shaking at 150 rpm for 2-3 days.

### Construction of the *Ntl *over-expression vector

An over-expression vector (pBarEx) for filamentous fungi was constructed based on pBTM. pBarEx contained a bar gene, promoter pGpdA, and terminator TTrpC from *A. nidulans *and a polylinker between pGpdA and TTrpC.

The full cDNA sequence of *Ntl *was amplified using Pyrobest DNA polymerase (TaKaRa, Japan) with primers B1 (5'-AAT TAC GCG TAC CTC CAC GTT CGT CAG TC-3' with an *MluI *recognition sequence at the 5' end) and B2 (5'-CGC CAC GCG TTT GAG AGG GCA ATT AAT CG-3' with an *MluI *recognition sequence at the 3' end). The PCR product and vector pBarEx were both digested with *MluI*, and then ligated using T4 DNA ligase (pBarEx-NTL, Figure 1A).

### Construction of the *Ntl *RNAi vector

A dual promoter RNAi vector for filamentous fungi was first constructed based on pBTM, which was reported previously [44], pDPB containing a selectable marker, the bar gene (resistance to ammonium glufosinate), polylinker, and two promotors in opposite direction (pGpdA and pTrpC from *A. nidulans*).

A fragment of the coding sequence of *Ntl *(310-745) was then amplified from *M. acridum Ntl *cDNA with primers A1 (5'-ATT AAC GCG TAG CAC AAG AAG ATA CCG ATG-3' with an *MluI *restriction site at the 5' end) and A2 (5'-TAT AAC GCG TCG CGC CAG GGA GCT GCT GGA CAT CTAG-3' with an *MluI *restriction site at the 3' end), which was designed according to the CQMa102 *Ntl *cDNA sequence (GenBank AY557612). The PCR product and vector pDPB were both digested with *MluI*, and then ligated using T4 DNA ligase (Takara, Japan) (pDPB-NTL) (Figure 1B).

### Transformation of *M. acridum*

Intact *M. acridum *CQMa102 conidia were transformed by microparticle bombardment (Model PDS-1000/He biolistic particle delivery system, Bio-Rad, USA). For bombardment, 50 μL of conidia suspension (10^9 ^conidia/mL) were placed in the center of a Petri dish. Plasmids pDPB-NTL and pBarEx-NTL were linearized with *BamHI *and bound to 0.6-μm diameter golden particles and then transformed into *M. anisoplia *by particle-mediated DNA delivery (Model PDS-1000/He biolistic particle delivery system, Bio-Rad, USA), according to St Leger [45]. Following bombardment, conidia were resuspended in 5 mL of MilliQ water. Aliquots of 200 μL were plated on Czapek's medium (3% saccharose, 0.2% NaNO_3_, 0.1% K_2_HPO_4_, 0.05% KCl, 0.05% MgSO_4_, 0.001% FeSO_4_) containing 200 μg/mL ammonium glufosinate and incubated at 27°C for 6-8 days. Transformants were confirmed by PCR amplification of bar gene. Post-transformation mitotic stability was evaluated according to the method in a previous report [46].

### Quantification analysis of *Ntl *transcript

Total RNA was isolated from mycelia using the Trizol reagent (Invitrogen, USA). The cDNA was synthesized from DNaseI-treated total RNA with an anchored oligo-dT primer following the manufacturer's protocol (Promega, USA). Real-time PCR was performed using the SYBR-Green PCR Master Mix kit (Bio-Rad) in a Light Cycler (Bio-Rad). A standard curve was made to optimize the amplification efficiency with the primer pairs L1 (5'-GCACAAGAAGATACCGATGGC-3') and L2 (5'-CGATCCACTGGGTTCTCATTTA-3'). *Gdpdh *encoding gly ceraldehyde-3-phosphate dehydrogenase was selected as an internal control, and the primers of 5'-AGATGGAGGAGTTGGTGTTG-3' and 5'-GACTGCCCGCATTGAGAAG-3' were used for it [47]. The cycling conditions were 95°C for 3 min followed by 45 cycles of 95°C for 10 sec, annealing at 59°C (*Ntl*) or 60°C (*Gdpdh*) for 10 sec. The relative expression level of the *Ntl *in *M. acridum *transformants compared to that in wild-type strain was determined with the comparative cycle threshold (C_T_) method [48]. Biological techniques were conducted in quadruplicate.

### Measurement of trehalose concentrations and trehalase activity

Trehalose levels in conidia were measured using a method modified from Foster *et al*. [28]. Conidia of both wild-type and *M. acridum *transformants were harvested from 14 day plates, washed with distilled water, resuspended in 500 μL of water, boiled for 20 min, and disrupted by vortexing with glass beads (0.5 mm). Cell debris was removed by centrifugation at 13,000 g for 5 min and the supernatant was stored at 0°C prior to trehalose assay. A 50-μL aliquot of the conidia lysis solution was added to 50 μL of 0.1 M sodium citrate buffer (pH 5.6). Duplicate samples were incubated with or without 10 μL porcine kidney acidic trehalase (Sigma, USA) overnight at 37°C. The reaction was stopped by boiling the sample for 10 min. Following centrifugation, the glucose concentration in the supernatant was assayed via a glucose assay kit (Bioscience, China).

To assay trehalase activity, 25 μL of the trehalase extraction solution were added to the trehalose solution containing 50 mM HEPS, and the mixture was incubated for 30 min at 37°C. The reaction was stopped by boiling the samples for 10 min, the samples were centrifuged, and the glucose in the supernatant was assayed using a commercial kit (Trinder, Sigma).

### Heat shock treatment

Conidia were prepared as described above. For the wet-heat shock test, conidia were suspended in 1 mL sterilized water. The suspension was vigorously shaken and filtered through cotton cloth and diluted to a concentration of 1 × 10^7 ^conidia·mL^-1^. Subsequently, the suspension was immediately placed in a stirred water bath at 45°C for 0.5, 1, 1.5, 2, or 2.5 hours. For the dry-heat shock test, conidia were dried in a desiccator containing silica gel until the moisture content was less than 5%. Dried conidia were maintained in an incubator oven at 65°C for 1, 2, 3, 4, or 5 hours, and then suspended in sterilized water (1 × 10^7 ^conidia·mL^-1^). The conidial suspensions maintained at 28°C were used as a control. Germinations were measured by plating 50 μL on 1/4SDA plates. After 24 hours incubation in the dark at 28°C, the germination rate was checked with a microscope (Motic, china) at 400× magnification. About 300 conidia were evaluated for germination from different areas in each plate. Inhibition time values for 50% germination (IT_50_) were used to estimate the conidiospore thermotolerance of *M. acridum *using DPS software [49].

### Bioassays

*Locusta migratoria *were reared in our lab under crowded conditions as previously described by He et al. [50]. Male and female insects were separated after adult emergence. Male adult locusts (2-3 days after eclosion) were used in the bioassay tests. A 5-μL solution of 2 × 10^6 ^conidia/mL of either wild-type *M. acridum *or transformants in cottonseed oil (Sigma) was applied to the locusts' head-thorax junctions. Treated locusts were separately confined in cages (20 × 20 × 20 cm) by 40 mesh, and kept at a temperature of 28°C with a 16:8 h (light:day) photoperiod. There were four replications of n = 30 locusts in each treatment. Mortality was recorded daily and lethal time values for 50% mortality (LT_50_) values were used to estimate the infectivity of *M. acridum *by DPS software [49].

### Statistical analysis

All samples and treatments were carried out in triplicate unless stated otherwise. Data were square root arcsine transformed before being subjected to analysis of variance (ANOVA) for a completely randomized design. The means were separated using Tukey's multiple range test, carried out using DPS software [47]. Statistical significance was established at p < 0.05.

## Authors' contributions

YX designed the study. YL, GP, YC, and YX wrote the manuscript. YL, GP, YC, and YX performed the experiments in this study. In particular, GP performed the data analysis and bioassay experiments, and YC participated in construction of the vector. All authors read and approved the final manuscript.
